# Do Elite Athletes Live Longer? A Systematic Review of Mortality and Longevity in Elite Athletes

**DOI:** 10.1186/s40798-015-0024-x

**Published:** 2015-08-13

**Authors:** Srdjan Lemez, Joseph Baker

**Affiliations:** School of Kinesiology and Health Science, York University, 4700 Keele Street, Toronto, M3J 1P3 ON Canada

**Keywords:** Athletes, Death, Elite, Longevity, Mortality

## Abstract

**Background:**

Understanding of an athlete’s lifespan is limited with a much more sophisticated knowledge of their competitive careers and little knowledge of post-career outcomes. In this review, we consider the relationship between participation at elite levels of sport and mortality risk relative to other athletes and age- and sex-matched controls from the general population. Our objective was to identify, collate, and disseminate a comprehensive list of risk factors associated with longevity and trends and causes of mortality among elite athletes.

**Methods:**

English language articles were searched using the Web of Science database. Keywords *athletes*, *death*, *elite*, *“high performance*” life expect*, *longevity*, *mortality*, *players*, *professional*, and *sport* were used to locate research articles. Seventeen additional articles were retrieved from reference lists found in these papers and a general web search. The inclusion criteria were the following: (1) publication year 1980 or later; (2) the study examined elite-level athletes; and (3) outcome data measured mortality/longevity trends and/or causes.

**Results:**

Fifty-four peer-reviewed publications and three articles from online sources met the criteria for inclusion. Baseball, football, soccer, basketball, and cycling had the most reported data on elite athletes’ lifespan longevities. A variety of mechanisms have attempted to explain mortality risk (e.g., handedness, playing position, achievement, etc.). Considerable support was found for superior longevity outcomes for elite athletes, particularly those in endurance and mixed sports.

**Conclusions:**

Future research into the mechanisms that may affect mortality risk is important for a better understanding of life expectancies in both eminent and non-eminent populations. Participation in elite sport is generally favorable to lifespan longevity.

**Key Points:**

- A majority of studies included in this review reported superior lifespan longevity outcomes for elite athletes compared to age- and sex-matched controls from the general population and other athletes.
- Several mechanisms within and between sports may have powerful effects on the overall lifespan longevities of players (e.g., type of sport, playing position, race, and energy system).
- Future research on mortality in elite athletes would benefit from more comprehensive statistical measures and reliable databases to determine potential mechanisms that may influence mortality trends and causes in both athlete and non-athlete samples.

## Background

### Rationale

Researchers have given considerable attention to the athlete development process (e.g., positive youth development through sport) (see [1]). Interestingly, insight into post-career outcomes is significantly limited. For instance, our knowledge of how participation in elite sport affects lifespan longevity is particularly incomplete. This lack of information about later phases of athletes’ lifespans may be attributable to several factors, such as the greater access to athletes during their competitive careers as well as the preponderance of participation- and performance-based theories focusing on elements related to understanding the antecedents of sporting success (e.g., [2]). Attaining a *complete* understanding of an athlete’s lifespan requires knowledge of the inherent complexity of relationships that link hereditary and environmental characteristics to developmental outcomes. In this study, we focus on mortality of previously elite athletes.

An important challenge to mortality research in sport is the lack of data on the health behaviors of athletes post retirement. Much of our current understanding of trends and causes in elite athlete mortality derives from what appear to be “one-off” studies by small teams of researchers. Past work examining this issue in depth appears to be limited, although the Finnish studies that examined lifespan longevities of former elite endurance, team, and power athletes who represented Finland between the years 1920 and 1965 are exceptions (e.g., [3–6]). In addition, views on elite athlete mortality are largely shaped by popular media sources, which may downplay tenets of the lifespan and create false perceptions of early mortality in athletes (e.g., MLB’s Tony Gwynn at 54 years [cancer], the NFL’s Junior Seau at 43 years [suicide], boxing’s Tommy Morrison at 44 years [AIDS], sailing’s Andrew Simpson at 36 years [drowning] and skiing’s Sarah Burke at 29 years [training fall]).

A recent meta-analysis completed by Garatachea et al. [7] indicated that elite athletes live longer than the general population, with an all-cause pooled standard mortality ratio (SMR) of 0.67 (95 % confidence interval [CI] 0.55–0.81; *P* < 0.001). Further, they found a lower risk of cardiovascular disease (CVD) and cancer in those who participated in high-performance sport, which emphasized the health benefits of exercise [7]. While their study makes an important contribution to our knowledge of longevity outcomes in elite sport, the restrictive sampling frame necessary for a meta-analysis excluded numerous studies that could inform our broader understanding of elite athlete health. For example, Garatachea et al. [7] included 10 studies of elite athlete longevity in their meta-analyses, but their inclusion criteria excluded studies that did not use SMR as a statistical measure of mortality (38 studies were excluded for this reason). As a result, studies with important information regarding longevity in elite athletes were excluded, such as greater longevity in Norwegian professional divers (hazard ratio [HR] = 0.79, 95 % CI 0.63–0.997) [8], and important mechanisms that may influence longevity, such as sex, as indicated by Olympic medal-winning females having greater longevity relative to Olympic medal-winning male athletes (HR = 0.61, 95 % CI 0.51–0.72) [9], and race, as indicated by African-American professional basketball players having a 77 % greater risk of death compared with white players (HR = 1.77, 95 % CI 1.35–2.32) [10]. Further, several studies which reported SMRs were not included in the meta-analysis (e.g., [11–15]). As such, there is an opportunity to grow our current limited understanding of longevity outcomes of elite athletes by examining a wider range of studies and sports through a systematic review.

Similarly, Teramoto and Bungum [16] completed a narrative review of mortality and longevity of elite athletes; however, a relatively small sample of 14 epidemiological studies was included. Their primary method of literature retrieval was through the PubMed (1950-) and Scopus (1960-) databases, using keywords *mortality*, *longevity*, *life expectancy*, *death*, and (*elite/professional*) *athletes* and *players*. While this literature search strategy was appropriate, a large number of studies on elite athlete mortality and longevity were published during or after their publication year of 2010 (e.g., [3, 8–10, 12, 13, 15, 17–31]). As a result, mechanisms such as race have since been validated as more consistent indicators of early mortality (e.g., [10, 19]). Currently, it may be premature to make conclusions about the long-term value of being a professional athlete considering the new evidence that has emerged from literature.

Although it appears that there are many unanswered questions concerning athletes’ lifespan longevities, Teramoto and Bungum [16] presented enough empirical evidence to determine some cross-sport and energy system trends. Teramoto and Bungum [16] found a trend towards endurance (e.g., long-distance runners) and mixed-sport (e.g., soccer) athletes having more favorable survival outcomes relative to power sport (e.g., weightlifters) athletes and the general population. Similarly, a meta-analysis performed by Löllgen et al. [32] examined 38 studies that measured physical activity and all-cause mortality in samples of physically active individuals (non-elite athletes) and reported an overall significant relationship between physical activity participation and lower all-cause mortality. Light and moderate intensity levels of activity were generally associated with a reduction in mortality, whereas training at high intensities was not required for the main prevention against all-cause mortality [32]. While Löllgen and colleagues [32] provided evidence of physical activity positively influencing lifespan longevity independent of age and sex, the relationship between participation in *elite sport* and longevity can enhance our understanding of the benefits of physical activity at the highest levels of competition in unique athletic cohorts.

Teramoto and Bungum’s [16] review suggested that the type and dose of elite sport participation may ultimately determine mortality risk. In addition, Teramoto and Bungum [16] highlighted the importance of considering elite athletes as a heterogeneous group with respect to mortality trends; differences between and within sports exist. In particular, the differences in health-related behaviors between and within sports may also create modifiable factors that are associated with longevity and mortality. As a result, overall mortality risk is explained by several modifiable factors, such as obesity and physical inactivity, and non-modifiable (unchangeable) factors, such as age and race, that are unique to athletes. For instance, Baron et al. [19] found an overall decrease in mortality in National Football Players (NFL) who were active between 1959 and 1988; however, defensive linemen had increased mortality from CVD and cardiomyopathy. Moreover, those with a playing time body mass index (BMI) of >30 kg/m^2^ had a significantly higher risk of CVD, which was also influenced by race/ethnicity [19]. Although the effects of playing position on lifespan longevity may be more pronounced in contact sports such as football, we cannot overlook the differences and nuances in health-related behaviors between and within sports that may influence lifespan longevities.

### Objectives

A subject such as death rates in professional athletes may be more susceptible to sensationalism when trends begin to emerge; therefore, it is essential that the data being disseminated are transparent and accurate. In this review, we consider the relationship between participation at elite levels of sport and mortality risk relative to other athletes and age- and sex-matched controls from the general population. Our objective was to extend the narrative review of Teramoto and Bungum [16] with a more comprehensive and up-to-date list of studies on mortality and longevity in previously elite athletes. More specifically, our aim was to advance knowledge in this area by collating athlete mortality/longevity literature that may help refine future analytic methods, form evidence-based models of athlete longevity, and determine whether elite-level participation in high-performance sport produces a lifespan longevity advantage. Similar to Teramoto and Bungum’s [16] research questions, we asked the following: (1) do elite athletes have superior longevity outcomes relative to the general population, and (2) which mechanisms and risk factors are associated with longevity and are potential precursors to early mortality?

## Methods

### Literature Search

A systematic review of literature was performed using the Web of Science database (1 January 1980–30 September 2014; see Tables 1 and 2). Web of Science was chosen as our primary citation index as it contained over 90 million records through its 7 online databases, which would have made searches into smaller citation indexes largely redundant (e.g., EMBASE contains over 28 million records). Further, given this review’s objective, Web of Science’s databases appeared to be the most relevant to our study (e.g., Science Citation Index Expanded). Keywords, including *athletes*, *death*, *elite*, *“high performance*” life expect*, *longevity*, *mortality*, *players*, *professional*, and *sport*, were used to locate research articles. While a full electronic search strategy for at least *one* database is recommended [33], we further located research articles by searching the references of records that were identified through our database search, in addition to performing a general web search through the Google Scholar search engine.

**Table 1:** Peer-reviewed elite athlete mortality literature (>1980; *n* = 54)

Sport/Country	Authors	*N*	Key finding	LE vs. GP
MLB^a^	Abel and Kruger [35] (2004)	6038	Significant differences in longevity related to handedness (*F*[2,6035] = 0.13) (death < 2001)	--
MLB^a^	Abel and Kruger [36] (2005)	2604	LE: ~4–5 years longer (*f* = 188.0, *df* = 1, 2,555, *P* < 0.001); (1900–1950 debut)	↑
MLB^a^	Abel and Kruger [37] (2005)	3573	Median post-induction survival for HOFs was 5 years shorter than for non-inducted players, 18 years (CI 15.0–21.0) vs. 23 years (CI 22.1–23.9) for matched controls (OR = 1.37, CI 1.08–1.73); (death ≤ 2002)	--
MLB^a^	Abel and Kruger [38] (2006)	4492	LE: 4.8 years longer (SD = ±15.0); career length increased longevity (*F* = 3.4 *df* = 3,4379; *P* < 0.02); (1900–1939 debut)	↑
MLB^a^	Abel and Kruger [39] (2007)	3760	Earlier career debut predicted earlier death (*F*[8,2898] = 7.78, *P* < 0.001); (death < 2006; 1900–1935 debut)	--
MLB^a^	Abel and Kruger [40] (2007)	3835	Players (*n* = 11) with positive initials (e.g., A.C.E.; 80.4 ± *SE* = 3.0) lived significantly longer (*F*[2904] = 3.7, *P* < 0.03, two-tailed) by 13 years than players (*n* = 30) with negative initials (e.g., D.E.D.; 67.0 ± *SE* = 3.1) or players (*n* = 864) with neutral initials (67.1 ± *SE* = 0.5); players with positive initials lived significantly longer than their matched controls and those with negative initials (*P* < 0.05); (died before 1950)	--
MLB^a^	Boren and Erickson [41] (1998)	28	The most common toxin that lead to death by poisoning was carbon monoxide; low overall risk (death 1889–1995)	--
MLB^a^	Fudin et al. [42] (1993)	1686	*In response to Halpern and Coren’s* [43] *study:* Left-handers (*n* = 235) had a *M* longevity of 64.46 years (SD = 15.34) right-handers (*n* = 1451), a difference of 38.57 days (*t*[1684] = 0.09, *P* > 0.05 for compared to 64.56 years (SD = 15.02) for years lived; Halpern and Coren [43] reported a difference of 244.72 days); (considered longevity data through 1978)	--
MLB^a^	Halpern and Coren [43] (1988)	1708	*M* age at death for right-handers was 64.64 years (*n* = 1472; SD = 15.5) and 63.97 years for left-handers (*n* = 236; SD = 15.4), a significant difference (*Z* = 6.63, *P* < 0.001)	--
MLB^a^	Hicks et al. [44] (1994)	5441	*In response to Fudin et al.’s* [42] *study:* Reliable data were confirmed for 3501 right-handers (64.3 %), 1199 mixed-handers (22.0 %) and 741 left-handers (13.6 %); the differences in *M* days of life were not significant in each group (F[2,5338] = .59, *P* = 0.55) and between just right- and left-handers (*t*[4240] = −1.08, *P* = 0.28; *M* longevity less for right-handers)	--
MLB^a^	Kalist and Peng [45] (2007)	2641	Greater longevity overall (SMR = 0.31); positive relationship between education and longevity (HR = 0.74, CI 0.56–0.977); black players had a HR 2.47 times greater than white players (1963–1996 debut)	↑
MLB^a^	Reynolds and Day [15] (2012)	14,360	Greater longevity rates throughout the last century (1900–1999 debut); (SMR = 0.87, CI 0.85–0.89; 1930–1999)	↑
MLB^a^	Saint Onge et al. [46] (2008)	6772	LE: ~5 years longer, compared to 20-year-old U.S. males; at 20 years, players from the Modern Era can expect to live 65.5 vs. 52.4 years and 58.3 years from the Early and Golden Eras, respectively (1902–2004 debut)	↑
MLB^a^	Smith [29] (2011)	--	*In response to Abel and Kruger’s* [40] *study:* No relationship between name initials and longevity (*P* > 0.05)	--
MLB^a^	Smith [30] (2011)	102	*In response to Abel and Kruger’s* [37] *study:* Corrected data shows that there is no statistically significant difference in the LE of HOF players (*Z* = 0.06, two-sided *P* = 0.952)	--
MLB^a^	Waterbor et al. [47] (1988)	985	Greater longevity overall (SMR = 0.94); longevity was correlated with position and in-game achievement (1911–1925 debut)	--
NBA^a^	Fafian Jr. [48] (1997)	2810	Greater longevity overall, particularly in guards (active 1946–1994)	↑
NBA^a^	Lawler and Lawler [25] (2011)	3647	Handedness was not significantly related to LE (HR = 0.92, CI 0.54–1.60; *P* = 0.76); (active 1946–2009)	--
NBA^a^	Lawler et al. [10] (2012)	3366	White players lived longer (~1.5 years) than black players (HR = 1.77, CI 1.35–2.32); LE: ~4 year longer overall (active 1946–2005)	↑
NFL^a^	Abel and Kruger [49] (2006)	1512	LE: 6.1 years longer (SD = ±11.9); career length (*F* = 4.7, *df* = 2,1504; *P* < 0.01) and BMI (*R*² = 0.01, *P* < 0.01) increased longevity (debut < 1940)	↑
NFL^a^	Baron et al. [19] (2012)	3439	Greater longevity overall (SMR = 0.53, CI 0.48–0.59); BMI, race, and position were associated with longevity (active 1959–1988)	↑
NFL^a^	Lehman et al. [12] (2012)	3439	Greater longevity overall (SMR = 0.53, CI 0.48–0.59); increased overall risk of neurodegenerative MR (SMR = 2.83–3.26, CI 1.36–5.21); elevated ALS (SMR = 4.31, CI 1.73–8.87) and AD (SMR = 3.86, CI 1.55–7.95) subcategories (active 1959–1988)	↑
NFL^a^	Selden et al. [50] (2009)^b^	--	Review of recent data suggests ↑ CVD risk factors in players, particularly linemen	--
Boxing	Baird et al. [18] (2010)	339	Decline in premature MR after 1983 (rounds reduced from 15 to 12); (death 1950–2007)	--
Cricket^c^	Aggleton et al. [51] (1993)	3165	There was a significant lifespan longevity difference (*t*[3163] = 2.77, *P* = 0.006, two-tailed) between right-handers (*n* = 2580; 65.62 years) and left-handers (*n* = 585; 63.52 years); left-handers were more likely to die prematurely in accidents or in warfare (players in the British Isles from 1864–1983)	--
Cricket^c^	Aggleton et al. [52] (1994)	5960	No significant relation between mortality and handedness (*P* = 0.3); left-handers were more likely to die from unnatural causes (*P* = 0.03, log hazard 0.37, CI 0.04–0.70), particularly warfare (*P* = 0.009, log hazard 0.53, CI 0.13–0.92); (born between 1840–1960; players in the British Isles from 1864–1992)	--
Cyclists^d^	Marijon et al. [27] (2013)	786	Greater longevity overall in Tour de France participants (SMR = 0.59, CI 0.51–0.68, *P* < 0.0001); reduction in neoplasms (SMR = 0.56, CI 0.42–0.72, *P* < 0.0001) and CVD (SMR = 0.67, CI 0.50–0.88, *P* = 0.004); (1947–2012 participants)	↑
Cyclists^d^	Morcet et al. [13] (2012)	514	Greater longevity overall (SMR = 0.50, CI 0.34–0.71); although higher MR in younger cyclists (active 1960–1990)	↑
Cyclists^d,e,f^	Sanchis-Gomar et al. [28] (2011)	834	LE: ~8 years longer for Tour de France participants (*P* < 0.05); (active 1930–1964)	↑
Diving^g^	Irgens et al. [8] (2013)	3130	Greater longevity overall (HR = 0.79, CI 0.63–0.997), although increased violent deaths (born 1950–1999)	↑
Golf^a^	Coate and Schwenkenberg [21] (2012)	313	LE: 5.4 years longer (won prize money 1980–2009)	↑
Golf^h^	Farahmand et al. [11] (2009)	300,818	Greater longevity overall (SMR = 0.60, CI 0.57–0.64); greatest longevity in most skilled players (SMR = 0.53, CI 0.41–0.67); (born >1920, registered < 2001)	↑
PL^i^	Pärssinen et al. [53] (2000)	62	Increased premature MR (SMR = 4.6, CI 2.04–10.45; *P* = 0.0002), suspected from prior anabolic steroid use (placed first–fifth in Finnish championships, 1977–1982)	↓
Rugby^j^	Beaglehole and Stewart [54] (1983)	822	LE of All Blacks is the same as GP, although LE (73.0 years) for non-Māori All Blacks was ~10 years longer (CI 71.8–74.5) relative to the Māori All Blacks (1884–1981)	=
Skiing^g^	Grimsmo et al. [22] (2011)	122	Total MR was 9 % lower after a 30-year follow-up (*P* = 0.04); (study participants, 1976–1981)	↑
Soccer^e^	Belli and Vanacore [55] (2005)	350	Similar observed and expected MR, although a ten-fold increase of ALS MR (SPMR = 1158, CI 672–1998) was present (active 1960–1996)	=
Soccer^k^	Koning and Amelink [23] (2012)	371	Greater longevity overall (*P* = 0.003); (active 1970–1973)	↑
Soccer^l^	Kuss et al. [24] (2011)	812	Cumulative observed survival was smaller than cumulative expected survival; therefore, male and female players had reduced longevity (RSR ≤ 1); (active 1908–2006)	↓
Soccer^e^	Taioli [56] (2007)	5389	Greater longevity overall (SMR = 0.68, CI 0.52–0.86), although increased risk for car accident (SMR = 2.23, CI 1.46–3.27) and ALS (SMR = 18.18, CI 5.00–46.55) death (active 1975–2003)	↑
SW^m^	Kanda et al. [57] (2009)	73	Deceased wrestlers had higher BMIs (OR = 1.08, CI 1.01–1.15) and winning percentages (OR = 1.29, CI 0.86–1.93); (active 1926–1989)	--
T and F^c^	Menotti et al. [58] (1990)	983	Greater longevity in males (SMR = 0.73; *n* = 700) and females (SMR = 0.48; *n* = 283); significant when analyzed together (*P* = 0.0296); (active > 1940)	↑
Mixed^a^	Abel and Kruger [17] (2010)	10,216	Decrease in longevity associated with names beginning with A–D; linear decrease for baseball and hockey players, but non-linear in football and basketball players; for each sport, A names lived longer than E–Z names, and D names had decreased longevities compared to E–Z names *F*(4,10,193) = 4.16, *P* = 0.002); football players lived the longest (72.3 ± 12.7) and baseball players lived the least years (70.8 ± 14.7); significant letter differences occurred between A and E–Z (HR = 0.80, CI 0.71–0.91, *P* < 0.001) and D and E–Z (HR = 1.16, CI 1.04–1.30, *P* < 0.01); (born 1875–1930)	--
Mixed	Bianco et al. [59] (2007)	715	Baseball (LE: 76; *n* = 154), basketball (LE: 78; *n* = 58), boxing (LE: 73; *n* = 81),football (LE: 77; *n* = 81), ice hockey (LE: 74.5; *n* = 130), swimming (LE: 74; *n* = 37), tennis (LE: 79; *n* = 83), track and field (LE: 75; *n* = 59), and wrestling (LE: 77; *n* = 32) HOFs had greater overall longevity (*P* < 0.05); (*M* = 76 years); (born 1860–1930)	↑
Mixed	Clarke et al. [20] (2012)	15,174	11,619 (76.6 %) male and 3555 (23.4 %) female Olympic Game medalists’ (1896–2010) LE was 2.8 years longer (RCS = 1.08, CI 1.07–1.10); endurance (RCS = 1.13, CI 1.09–1.17) and mixed (RCS = 1.11, CI 1.09–1.13) sport athletes had a more favorable survival advantage relative to power sport athletes (RCS = 1.05, CI 1.01–1.08)	↑
Mixed^a^	Coate and Sun [9] (2013)	2690	Females (*n* = 1348; HR = 0.61, CI 0.51–0.72) had a ~6 year LE advantage over males (*n* = 1342), even though they competed in the same Olympic events (1900–2008) and tennis championships (HR = 0.65, CI 0.47–0.91; >1880); Olympic medalists’ LE: 2–3 years longer if born <1920, but smaller advantage overall; tennis players’ LE: 5–6 years longer if born <1920, and 2–3 years overall; *M* LE was 82 years for females and 76 years for males	↑
Mixed^n^	Gajewski and Poznańska [60] (2008)	2113	Greater longevity overall in male (*n* = 1689; SMR = 0.50, CI 0.44–0.56) and female (*n* = 424; SMR = 0.73, CI 0.48–1.05) Olympians (1924–2000)	↑
Mixed^i^	Kettunen et al. [3] (2014)	2363	LE: ~5–6 years longer overall; endurance (79.1 years; CI 76.6–80.6), team (78.8 years; CI 78.1–79.8) and power (75.6 years; CI 74.0–76.5) sport athletes who represented Finland in international competitions (active 1920–1965) had higher LE than controls (72.9 years, CI 71.8–74.3), even after adjusting for socio economic status, birth cohort, and disease-specific mortality (HR ≤ 1); boxers had increased risk of dementia mortality (HR = 4.20, CI 2.30–7.81)	↑
Mixed^i^	Kujala et al. [4] (2001)	2009	Greater longevity overall (SMR = 0.74, CI 0.69–0.79); all-cause mortality was lower for endurance (SMR = 0.57, CI 0.47–0.68), mixed (SMR = 0.68, CI 0.61–0.76), and power (SMR = 0.90, CI 0.81–1.00) sport athletes who represented Finland in international competitions (active 1920–1965); increased risk of hypertension in power sport athletes (SMR = 2.63, CI 1.06–5.42); (SMRs calculated 1971–1995)	↑
Mixed^h^	Lindqvist et al. [26] (2013)	1199	MR was not increased overall in former power sport athletes, except a slight increase at 45 years (CI 2.1–4.2); between 20 and 50 years, estimated HR = 1.44–1.46; 2.1–3.9 times increased MR from suicide between 30 and 50 years (overall, HR = 1.74, CI 1.08–2.66; *P* = 0.025); however, there was a lower malignancy MR (HR = 0.71, CI 0.50–0.98; *P* = 0.036); (active 1960–1979)	--
Mixed^n^	Poznańska and Gajewski [14] (2001)	--	Greater overall longevity (SMR = 0.42, CI 0.35–0.49), particularly between 1992 and 1998 (Olympians 1981–1998)	↑
Mixed^b^	Samaras et al. [61] (2002)	1505	Modest correlation coefficients indicated that weight is only one risk factor that affected the longevities of baseball players (*n* = 1278; *r* = −0.22, *P* < 0*.*025) [58], football players (*n* = 199; *r* = −0.33, *P* < 0*.*005) [59], and Finnish elite athletes (*r* = −0.51, non-significant) [5]	--
Mixed^i^	Sarna et al. [5, 6] (1993, 1997)	2613	LE for endurance (5.7; *M =* 75.6, CI 73.6–77.5), team (4.0*; M =* 73.9, CI 72.7–75.1), and in power (1.6; *M =* 71.5, CI 70.4–72.2) sport athletes was greater than the referents (69.9); decreased CVD in endurance (OR = 0.49, CI 0.26–0.93) and team (OR = 0.61, CI 0.41–0.92) sport athletes (active 1920–1965)	↑
Mixed	Zwiers et al. [31] (2012)	9889	High-intensity sport athletes had lower longevity compared to low-intensity sport athletes (high risk of bodily collision, HR = 1.11, CI 1.06–1.15, and high levels of physical contact, HR = 1.16, CI 1.11–1.22 (Olympians 1896–1936; sex breakdown unknown)	--

Studies with no subscript analyzed multiple countries, or other sports were used as controls. Full citations provided in reference list; all LE and MR data were compared to age-matched controls from the GP and all studies were on male participants, unless stated otherwise; descriptions reflect only the key findings

*AD* Alzheimer’s disease, *ALS* amyotrophic lateral sclerosis, *BMI* body mass index, *CI* confidence interval, *CVD* cardiovascular disease, *GP* general population, *HOF* hall of fame, *HR* hazard rate/ratio of death, *LE* life expectancy, *M* mean, *MLB* Major League Baseball, *MR* mortality rate, *NBA* National Basketball Association, *NFL* National Football League; *OR* odds ratio for mortality, *PL* powerlifting, *R²* explained variation/total variation (coefficient of determination), *RCS* relative conditional survival, *RSR* relative survival ratio, *SD* standard deviation, *SMR* standardized mortality ratio, *SPMR* standardized proportionate mortality ratio, *SW* sumo wrestling, *T and F* track and field

^a^USA

^b^Reviews met inclusion criteria

^c^United Kingdom

^d^France

^e^Italy

^f^Belgium

^g^Norway

^h^Sweden

^i^Finland

^j^New Zealand

^k^Netherlands

^l^Germany

^m^Japan

^n^Poland

**Table 2:** Online elite athlete mortality articles (*n* = 3)

Sport/Country	Authors	*N*	Key finding	LE vs. GP
NFL^a^	Baron and Rinsky [62] (*NIOSH*) (1994)	6848	NFL players had a 46 % decreased MR (SMR = 0.54); linemen had a 52 % greater risk of death from heart disease than the GP, and three times the risk compared to football players; players had a decreased risk of death from violence (79 %) and accidents (39 %); (players since 1959; death through 1991)	↑
NFL^a^	Hargrove [63] (*Scripps Howard News Service*) (2006)	3850	The heaviest NFL players were more than twice as likely to die before their 50th birthday than their teammates; players are generally not dying sooner than average, but offensive and defensive linemen had a 52 % greater risk of dying from heart disease than the GP; out of the 130 players who died before age 50, 1/69 players born since 1955 are dead, 22 % of which died of heart diseases (77 % qualified as obese) and 19 % from homicides or suicides; when compared to *2403 MLB* players who have died in the last century, NFL players are more than twice as likely to die before age 50 (born since 1905)	--
Mixed^a^	Barnwell [64] (*Grantland*) (2012)	4582	12.8 % of football players had died (*n* = 3088) compared to 15.9 % of baseball players (*n* = 1494) as of 2007 (active from 1959–1988)	--

*AD* Alzheimer’s disease, *ALS* amyotrophic lateral sclerosis, *BMI* body mass index, *CI* confidence interval, *CVD* cardiovascular disease, *GP* general population, *HOF* hall of fame, *HR* hazard rate/ratio of death, *LE* life expectancy, *M* mean, *MLB* Major League Baseball, *MR* mortality rate, *NBA* National Basketball Association, *NFL* National Football League; *OR* odds ratio for mortality, *PL* powerlifting, *R²* explained variation/total variation (coefficient of determination), *RCS* relative conditional survival, *RSR* relative survival ratio, *SD* standard deviation, *SMR* standardized mortality ratio, *SPMR* standardized proportionate mortality ratio, *SW* sumo wrestling, *T and F* track and field

^a^USA

### Inclusion Criteria

The inclusion criteria were the following: (1) publication year 1980 or later; (2) the study examined elite-level athletes; and (3) outcome data measured mortality/longevity trends and/or causes. We excluded studies with no full-text availability (abstracts, conference proceedings, commentaries, and editorials), no English-text availability (a small proportion of international articles were unavailable for translation to English), other literature reviews with different inclusion criteria (e.g., non-elite samples), duplicates, case reports, studies on morbidity (i.e., CVD risk factors, etc.), psychosocial measures, collegiate athletes, and studies on the effectiveness of pre-screening strategies and prevention. For consistency, we use “elite” synonymously with any form of high-performance participation in sport (i.e., national, professional, and international competition such as the Olympics).

### Data Extraction

**Fig. 1:**
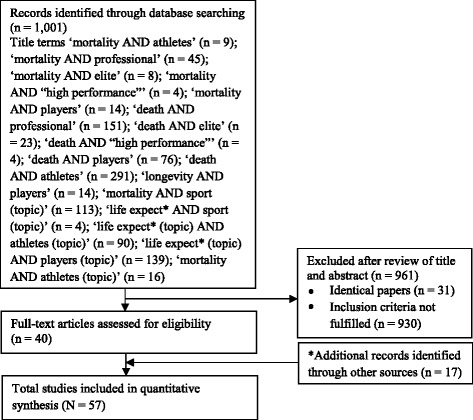
Flow of information through the different phases of the systematic review, as per the PRISMA statement [33]. *Additional records were identified through searching the references of records that were identified through database searching and a general web search (i.e., Google Scholar)

We identified 1001 records through database searching using the aforementioned keywords. After review of title and abstract, we excluded 961 of those records (identical papers, *n* = 31; inclusion criteria not fulfilled, *n* = 930; most commonly due to studies not examining elite athletes and/or mortality/longevity trends), which left 40 eligible full-text articles from the Web of Science database search. Seventeen additional articles were retrieved from reference lists found in these papers and a general web search. In total, 57 studies filled the criteria for inclusion (Tables 1 and 2): 54 peer-reviewed publications (see Table 1), in addition to three articles from online sources included to investigate findings of mortality risk in elite athletes that may be disseminated to a different cohort of the population (e.g., social media users; see Table 2). Specifically, these three additional articles were located through Google Scholar’s search engine using the same keywords as used in the Web of Science database search (e.g., athletes, death, etc.). We assessed the quality of each of these records through the Newcastle-Ottawa Quality Assessment Scale for cohort studies [34]. See Fig. 1 for the PRISMA statement [33].

### Measured Outcomes

This review was comprised of elite athlete mortality/longevity studies from 13 different countries: USA, France, Italy, Belgium, Norway, Sweden, Finland, New Zealand, Netherlands, Germany, Japan, Poland, and the UK. Measures of mortality included hazard rate/ratio of death (HR), life expectancy (LE), mortality rate (MR), odds ratio for mortality (OR), relative conditional survival (RCS), relative survival ratio (RSR), standardized mortality ratio (SMR), and standardized proportionate mortality ratio (SPMR). All LE and MR data were compared to age-matched controls from the general population and all studies were on male participants, unless stated otherwise in the tables.

## Results

### Literature Search

From 1 January 1980 to 30 September 2014, we located 54 peer-reviewed studies [3–6, 8–15, 17–31, 35–61] and three online articles [62–64] that examined mortality and longevity in elite athletes (*n* = 57). This is an addition of 43 peer-reviewed studies that were not included in Teramoto and Bungum’s [16] review (11 peer-reviewed studies overlap). Three peer-reviewed studies from their review did not match our inclusion criteria (<1980).

### Summary of Life Expectancy in Elite Athletes from Literature

This review included a total of 465,575 athletes: 450,295 from peer-reviewed literature (Table 1) and 15,280 from online articles (Table 2). Of those 465,575 examined, only 5610 (1.2 %) were female athletes. Although it appears that females have been grossly underrepresented in mortality and longevity studies of elite athletes, the extremely low proportion of 1.2 % is skewed by separate studies examining the same cohort of players [e.g., 12 and 19, *n* = 3439; 3–6, active Finnish athletes from 1920 to 1965), single studies that contained very large sample sizes of male athletes [e.g., 11; *n* = 300,818), and instances where the breakdown of sex in the sample went unreported (e.g., [31]). Nevertheless, only 6 peer-reviewed studies on elite athlete mortality and longevity that included females in their samples were identified [9, 20, 24, 31, 58, 60] and no studies that investigated females exclusively.

From the 9-point Newcastle-Ottawa Quality Assessment Scale [34], 1 study had a quality score of 6, 6 studies had a quality score of 7, 32 studies had a quality score of 8, and 15 studies had a quality score of 9 (only peer-reviewed articles were assessed). Therefore, the majority of the studies included in this review were generally of high quality (e.g., representative sample sizes, age- and sex-matched control groups from the general population, etc.).

Of the 54 peer-reviewed studies included, 4 were responses to the authors of different studies related to mortality and longevity of elite athletes [29, 30, 42, 44]. Aside from the studies or reviews that examined multiple sports, professional baseball players (*n* = 16), football players (4 peer-reviewed, 2 online), soccer players (*n* = 4), basketball players (*n* = 3), and cyclists (*n* = 3) were identified through our literature search as having the most reported data on elite athletes’ mortality outcomes. In particular, MLB players [15, 36, 38, 45–47], NFL players [12, 19, 49, 62], cyclists [13, 27, 28], NBA players [10, 48], and golfers [11, 21] had the most robust evidence of greater longevity. These LE benefits generally ranged from 4 to 8 years [e.g., 36 and 28, respectively].

The majority of studies included in this review examined sport organizations that were primarily located in the USA and used age- and sex-matched controls that were also American (MLB, NBA, and NFL). The general finding of these studies was a greater longevity for elite athletes relative to their American controls. Notably, the majority of mixed-sport studies included in this review also found support for greater longevity for athletes who competed at elite levels of competition (e.g., Olympics). Similarly, the “one-off” studies from international researchers whose controls spanned 12 different countries also reflect a general trend towards increased survival rates for a diverse group of athletes relative to their country-specific controls from the general population. For example, elite cyclists, such as French, Italian, and Belgian Tour de France participants, had greater lifespan longevities when compared to the pooled general population from their respective countries for the appropriate age cohorts [13, 27, 28]. Further, Norwegian divers [8] and skiers [22] and Italian track and field athletes [58] had greater lifespan longevities relative to controls from their respective countries as well. Alternatively, less consistent results were found in soccer players, in which both superior survival rates in Dutch players [23] and inferior survival rates in German players [24] were reported, alongside increases in amyotrophic lateral sclerosis (ALS) prevalence in Italian players [55, 56]. In addition, elite Finnish powerlifters [53] displayed lower lifespan longevities compared to the Finnish general population.

### Mechanisms of Mortality and Primary Outcomes

Considerable research attention has been given to identifying which mechanisms may be precursors to early mortality, including handedness, precocity, names and initials of players/athletes, playing position and weight, education and race, achievement, and energy system classifications. First, the relationship between handedness and mortality in elite athletes has received increased attention within the last two decades. Nevertheless, differences in longevity related to handedness appear to be non-existent in MLB [35, 42, 44] and NBA players [25]. While a relationship between longevity and handedness was identified in elite cricketers [51], the inclusion of a larger sample size appears to have washed out previous significant findings [52]. Second, the precocity-longevity hypothesis (see [65]), which investigates the relationship between precociousness (i.e., career debut) and early death, has been shown to affect MLB players’ longevities [39]. Third, MLB players with positive initials in their names (e.g., A.C.E.) were found to live significantly longer than players with negative initials (e.g., D.E.D.) [40] while baseball, hockey, football and basketball players with names beginning with D had shorter lifespans than those with names beginning with E to Z [17]. It is noteworthy that there has been some criticism regarding the methodologies used in these studies, such as using selective data and the appropriateness of the statistical tests performed [29, 30]. Further, while it has been suggested that health is influenced by certain symbolic aspects of the environment [66], such as a decreased prevalence of death before birthdays (e.g., [67]), the scientific rationale behind the initials of a name affecting lifespan longevity is limited. In turn, the emergence of a hypothetical name-longevity relationship in elite athletes emphasizes the need for greater replication in this area of research.

An athlete’s playing position is arguably the most obvious mechanism that influences mortality risk, largely due to perceived anthropometric differences that are determinants of health (e.g., weight). Longevity was found to be correlated with position in MLB [47] and NBA players [48]. In particular, *weight* and position significantly influenced mortality risk in NFL players [19, 49, 50, 61–63, 68]. Further, weight also influenced the lifespan longevities of baseball players [61, 69] and played a role in the longevities of Japanese sumo wrestlers, although its influence appeared to be small [OR = 1.08, CI 1.01–1.15; 57]. With regard to athlete race, this has been shown to be associated with longevity in NBA [10] and NFL players [19], as well as with education and longevity in MLB players [45]. Sixth, high achievement in sport emerged as a determinant of mortality, specifically through winning percentage in Japanese sumo wrestlers [57], handicap in Swedish golfers [11], and Hall of Fame (HOF) induction in a diverse range of sports [37, 59]. Although Abel and Kruger [37] reported differences in the life expectancies of MLB HOFs compared to non-inductees, Smith [30] did not find a statistically significant difference using corrected data from the same sample.

The last trend that developed from these studies, which served as a classification method for Teramoto and Bungum’s [16] review, was the relationship between the type of sport and required energy systems for participation (i.e., aerobic/endurance, mixed, and anaerobic/power) and mortality. Similar to Teramoto and Bungum’s [16] findings, the largest gains in lifespan longevities were in endurance and mixed-sport athletes. The greatest LE advantages were found in European cyclists [13, 27, 28], whereas the lowest LEs were found in Finnish powerlifters [53]. Cross-sport analyses uniformly reported LE advantages in endurance and mixed-sport athletes compared to power sport athletes [3–6, 20, 31], who had some evidence of increased premature mortality from suicide suspected from prior anabolic steroid use [26, 53]. The inconsistent mortality outcomes in power sport athletes make it difficult to generalize across all sports. Nevertheless, there was considerable support in the existing literature for superior longevity outcomes for elite athletes compared to the age- and sex-matched controls from the general population.

## Discussion

The objective of our review was to advance knowledge on elite athlete mortality and longevity to ultimately determine whether elite-level participation in high-performance sport produces a lifespan longevity advantage. As a whole, the empirical evidence suggests that several mechanisms within and between sports have powerful effects on the overall lifespan longevities of players. Our first research question considered whether elite athletes had superior longevity outcomes relative to the general population. An overwhelming majority of studies included in this review reported favorable lifespan longevities for athletes compared to their age- and sex-matched controls from the general population. In fact, only two studies reported lower lifespan longevities in athletes relative to the controls: 812 male and female soccer players who participated in international matches for Germany between 1908 and 2006 (RSR ≤ 1) [24], and 62 male Finnish powerlifters who placed first–fifth in their respective weight category in the Finnish championships between 1977 and 1982 (SMR = 4.6) [53]. Our second research question explored the mechanisms and risk factors associated with longevity and whether there were precursors to early mortality. Although our overall understanding of modifiable and non-modifiable factors that contribute to mortality risk in elite athletes remains limited, in part due to methodological and data source inconsistencies [29, 30], some trends emerged from our investigation. In particular, our review supports previous conclusions that aerobic and mixed-sport athletes have superior longevity outcomes relative to more anaerobic sport athletes. In addition, playing position and weight, as well as education and race, appeared to be consistent indicators of mortality risk, whereas other mechanisms such as handedness, precocity, and names and initials appeared to be less consistent and/or examined.

In their review of the relationship between body size and lifespan longevity, Samaras et al. [61] drew attention to several confounders that may positively influence longevity outcomes, such as higher socioeconomic status, smaller body size, and positive environmental and health benefits. As highlighted in this review, weight is an important predictor of mortality risk. Likewise, significant empirical evidence suggests that obesity is one of the major risk factors for premature death (e.g., [70]). While it is premature to undervalue the relationship between weight and mortality in athletes, particularly post-retirement, research has also highlighted the importance of accounting for body composition. For example, it has been suggested that a measure of BMI is likely to overestimate adiposity in muscular athletes (e.g., [71]), particularly in NFL players [19]. In addition, Koning and Amelink [23] highlighted that self-selecting to participate in an occupation where health is important may predispose athletes to favorable survival outcomes relative to population comparisons. Factors such as these, in addition to a bevy of other confounders such as access to high-quality medical care [23], are what make LE a difficult outcome to accurately predict.

As a variety of confounders may impact longevity, the reasons for the differences in lifespans between elite athletes and the general population are likely to be multifactorial. Teramoto and Bungum [16] provided possible explanations of increased survival in the elite athlete cohort; namely, participation in higher volumes of exercise training leading to higher physical fitness levels, the likelihood that elite athletes are comprised of the healthiest and fittest individuals, and the maintenance of active and healthy lifestyles later in life. The extents to which these confounders contribute to mortality risk are still largely unknown however, as survival statistics may undermine the interplay of complex socioeconomic factors [72]. For example, medical care accessibility made available by higher income may improve the LE of athletes when compared to other groups. Further, plenty of corroborating evidence suggests health-care services alone do not result in improved health outcomes, but a variety of social factors such as education and employment produce these widespread biases in health (e.g., [73]). As a result, the historical investigations of elite athletes and longevity outcomes need to be cautiously interpreted and discussed in the contexts of a variety of possible influential factors of mortality.

Abel and Kruger [17] discussed two advantages to studying elite athletes with regards to longevity. First, they represent a relatively homogenous occupational population, similar to Teramoto and Bungum’s [16] classification of a distinct physically fit and healthy group, and, perhaps more importantly, many sports contain detailed statistical and historical databases that track a variety of variables that can influence longevity (e.g., anthropometrics, performance-based indicators such as induction into the HOF, etc.). These advantages help contribute to the growing body of research on elite athlete mortality trends, which in turn can advance research by forming evidence-based models of athlete longevity through investigations into a variety of variables. In contrast, a caveat to this tendency of measuring the effects of sport-specific variables on mortality is that the amount of data available varies from sport to sport and may be somewhat arbitrary. For example, handedness is unlikely to be measured in soccer players, and the influence of position in non-contact sports may be more relevant in life quality research rather than life longevity. In addition, each sport will have a different inaugural season, which limits the amount of deceased players in the relatively newer sports (e.g., mixed martial arts). Therefore, each sport will have unique statistical variables that may make it more difficult for researchers to draw cross-sport comparisons.

## Limitations

The main finding of this systematic review indicates favorable lifespan longevity advantages for elite athletes relative to age- and sex-matched controls from the general population; however, limitations in reviewing literature on mortality and longevity in elite athletes exist. We will first examine the possible biases in epidemiological research in historical samples of athletes.

### Databases

The use of accurate and up-to-date databases is extremely important when analyzing a sample longitudinally; however, some statistical databases for past players have been found to be incomplete. For example, Smith [30] discovered a substantial portion of missing death date data (e.g., unknown death dates) in former MLB players in the Sean Lahman Baseball Archive [74]. Unfortunately, Smith’s [30] critique of Abel and Kruger’s [37] conclusion (cases without death date data were treated as living players) that HOF non-inductees had a 5-year lifespan longevity advantage was not the only study on MLB player longevity that used the same database (e.g., [17, 35, 36, 38–40, 45]). Although it may be premature to conclude that other databases have similar fallibility (e.g., missing death date data), we must be cautious of the possible incongruency between reported and unreported/unknown death date data in other studies that bias lifespan longevity results.

### Holistic Health

As a variety of socioeconomic, demographic, and epidemiologic factors dynamically interact to shape population change [72], so do factors that influence holistic health. Arguably, the most objective measurement of elite athlete health is rate of mortality. Using mortality statistics of elite athletes who played in the earlier decades to make inferences regarding holistic health in present-day athletes may be deceptive. The information age has made information on elite athletes easy to access, whereas information on elite athletes from the earlier eras often fails to depict the mental and social wellbeing characteristics that encompass holistic health. Sorenson et al.’s [75] investigation of lifespan exercise among elite intercollegiate athletes is one of few studies that have presented empirical evidence of lifetime health and wellbeing in modern competitive athletes relative to age- and sex-matched controls. They found that current student athletes reported higher volumes of weekly exercise, perceived exercise importance, and likelihood of compliance with American College of Sports Medicine (ACSM) exercise guidelines relative to non-athletes [75]. Interestingly, Sorenson et al. [75] found no significant differences between alumni student athletes and non-athletes, suggesting that former athletes failed to maintain higher exercise levels later in life. Further, in their follow-up study on the same sample, Sorenson et al. [76] found that relatively older former student athletes (age 43+ years) had a greater risk for joint health concerns later in life compared to a non-athlete control group. These findings seem counterintuitive since physical activity is often associated with a substantial reduction of chronic disease risk and being important to overall health and wellbeing (e.g., [77]).

To broaden our knowledge on holistic health outcomes and behavior in former athletes, it is important to consider the totality of data that have been collected and analyzed to date, particularly as research pertaining to the “whole person” (e.g., physical and psychosocial measures) in modern competitive athletes gains momentum. It is equally important to make the distinction between “quality of life” and “longevity,” as physical health likely moderates psychosocial health. For example, evidence suggests that physical activity plays an important role in managing mental health diseases, such as anxiety and depression (e.g., [78]). Thus, factors such as being physically capable to participate in physical activity are important to consider when determining an individual’s quality of life. As such, our current understanding of elite athletes’ quality of life during and after sport is limited relative to their lifespan longevity trends.

### Statistical Measures

Another criticism of the athlete-mortality literature is on methodological grounds; more specifically, cross-study discrepancies in the statistical tests and/or measures used. Although the relative paucity of lifespan longevity studies of elite athletes may serve as a temporary explanation for why different measures and control variables are used to analyze mortality, we cannot ignore the impact of possible statistical bias risk. Risk of bias can affect the cumulative evidence of a review of literature [33], such as selective reporting within studies, whereby researchers may under-report variables that were found to have less statistical impact on longevity outcomes in the course of reporting results that support the direction of their findings. As a result, the extent of our knowledge about the influence of certain variables on longevity may be restricted. Future work in this area of research would benefit from replication of control variables when analyzing the same or similar athletic populations to better establish important predictors of longevity. Further, meta-analyses on the longevity of elite athletes, such as the review completed by Garatachea and colleagues [7], can provide more evidence-based data on the benefits of participating in physical activity. Moreover, it is notable that the sports examined have examined periods of different length. Ideally, comparisons would be best when comparing timespans of similar length; however, the timespans investigated have ranged from players being born from as early as 1840 [52] to being active as recently as 2012 [27]. These differences could affect the proportion of those living or dead in a sample, which may affect measures such as SMR. The implication of these biases on the cumulative evidence of this review is unclear, and as a result, it is important to consider how publication bias can under- and overestimate certain predictors of longevity.

### Longitudinal Lifestyle Factors

Given that empirical evidence is necessary for coherent explanations of longevity outcomes of elite athletes, do mechanisms that influence mortality become ineffective and/or less powerful if maintenance of physical activity ceases? Although there has been some evidence that former intercollegiate student athletes fail to maintain higher exercise levels later in life [75], our understanding of the impact of different longitudinal lifestyle factors influencing lifespan longevity remains incomplete. For example, former male Finnish world class athletes were found to be more active than their non-competitive controls [79], and participation in physical activity at a young age predicted later life involvement, which reduced the prevalence of coronary heart disease [80]. In addition, former athletes have been found to partake in fewer negative health habits, such as smoking and drinking alcohol [79].

Another issue that is emerging from more recent research relates to the possible detrimental effects of high levels of training. In particular, O’Keefe (e.g., [81, 82]) has advocated that “excessive” aerobic training can result in cardiovascular damage (e.g., atrial fibrillation, coronary artery disease, and malignant ventricular arrhythmias). These effects may have particular relevance for studies of mortality in previously elite athletes. It is important for future research to determine which factors are more robust predictors of longevity and if they continue to be relevant in later life. In turn, these findings will have implications on the generalizability of factors found to predict mortality and longevity in elite athletes that were measured at one point in time (i.e., active athletic career).

## Future Directions

The relationship between sport and health has evolved considerably over the past 100 years. The context in which the historical data are transferable is important to consider, particularly when discussing the social determinants of health [73]. Despite its limitations, historical analyses of sport and health shape our present understanding of its relationship and influence.

### Reliable Databases, Repetition, Causes of Mortality, and Follow-up Studies

Continued contributions to the growing body of research on longevity outcomes of elite athletes should utilize appropriate statistical testing with reliable and complete databases. Although there are a variety ways to statistically measure and report mortality, research must be substantiated through repetition. An important first step is locating or comprising a reliable and comprehensive database that embodies all accessible and applicable data. To this end, future research of athlete lifespan outcomes can arguably have the greatest impact by determining the *causes* of mortality. Current empirical evidence on the rates of mortality in athletes is far superior to our knowledge on the causes of mortality. Epidemiological studies with long-term follow-ups are also rare [55]. For example, available evidence suggests a possible connection between dietary supplements and/or drug use and the high prevalence of amyotrophic lateral sclerosis (ALS) in former soccer players [55, 56]. This potential association emphasizes negative long-term neurological outcomes of performance demands that are not reflected in MR occurrence.

### Cross-sport Comparisons and Generalizability

Several sports were noticeably absent from the elite athlete mortality literature (e.g., ice hockey, field hockey, handball, snowboarding, table tennis, volleyball, and motorsports). In addition, sex-related differences in lifespan longevity remain largely unknown due to the paucity of studies on elite female athletes. This raises an important concern about the cross-sport generalizability disseminated in this review. Perhaps more importantly, the applicability of these results to the general population (i.e., non-elite-athletes) can likely be best explained by mechanisms of mortality that have not been extensively examined to date in the athlete cohort. Some examples include smoking and diet/nutrition (as stated by Teramoto and Bungum) [16], lifespan health (psychosocial and physical, such as the influence of morbidity on life quality, etc.), and the interplay of nature (hereditary, such as superior genotypes for physical fitness) (e.g., [83]) and nurture (environmental influences). Presumably, elite athletes possess advantageous genetic traits. Research on the heritability of physical fitness (e.g., [84]) suggests that we cannot discount the influence of advantageous genetic inheritance coupled with high levels of participation and competition in sport.

## Conclusions

Mechanisms such as type of sport, playing position, weight, education, and race can inform our understanding of lifespan longevity, which places increased responsibility on future research to demystify and contextualize mortality risk in both eminent and non-eminent populations. In conclusion, while additional research studies are needed to address quality of life and wellness outcomes, this review highlights mortality trends among elite athletes and concludes that participation in elite sport is generally favorable to lifespan longevity.

### Ethical Standards

This manuscript does not contain clinical studies or patient data.
